# Supplementary material for: Cinnamal Sensing and Luminescence Color Tuning in a Series of Rare-Earth Metal−Organic Frameworks with Trans-1,4-cyclohexanedicarboxylate
Source: Molecules. 2021 Aug 25;26(17):5145. doi: 10.3390/molecules26175145 (PMC8433715; doi:10.3390/molecules26175145)
Supplement: Supplementary file 1 [file molecules-26-05145-s001.zip › molecules-1343155-supplementary.pdf]

Cinnamal Sensing and Luminescence Color Tuning in a Series of Rare-Earth Metal−Organic Frameworks with Trans-1,4-cyclohexanedicarboxylate

Pavel A. Demakov [1,*], Alena A. Vasileva [1,2], Sergey S. Volynkin [1], Alexey A. Ryadun [1], Denis G. Samsonenko [1], Vladimir P. Fedin [1], Danil N. Dybtsev [1]

[1] Nikolaev Institute of Inorganic Chemistry, Siberian Branch of the Russian Academy of Sciences, Novosibirsk 630090, Russia

[2] Novosibirsk State University, 2 Pirogova St., Novosibirsk 630090, Russia

* Correspondence: demakov@niic.nsc.ru

Supplementary Information

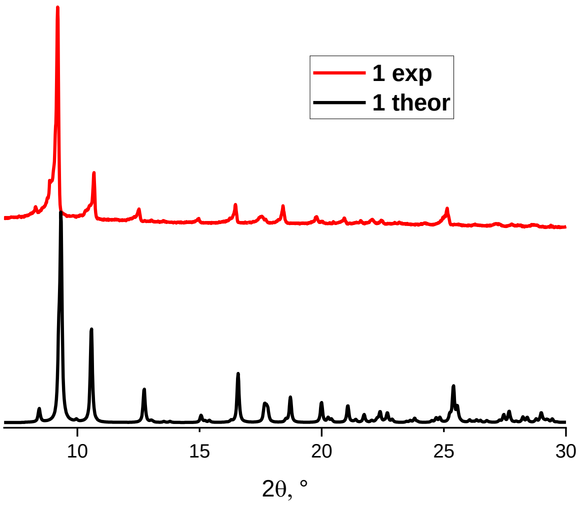

Figure S1. Experimental PXRD pattern for **1** compared to the theoretical one.

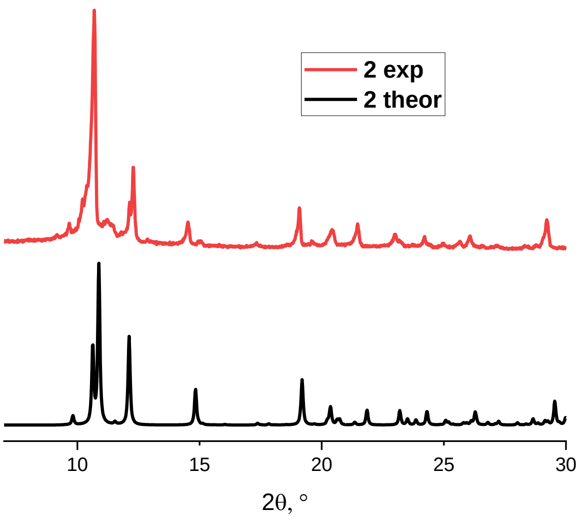

Figure S2. Experimental PXRD pattern for **2** compared to the theoretical one.

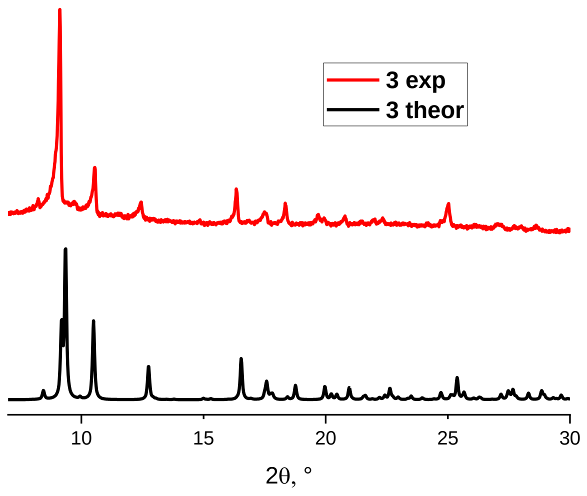

Figure S3. Experimental PXRD pattern for **3** compared to the theoretical one.

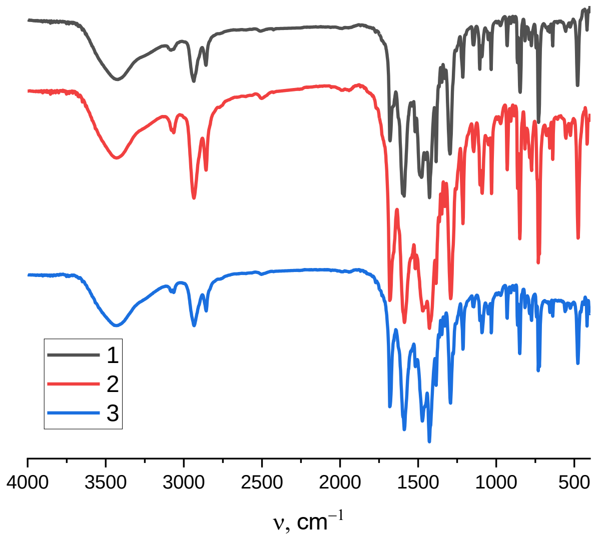

Figure S4. IR spectra for **1 – 3**

.

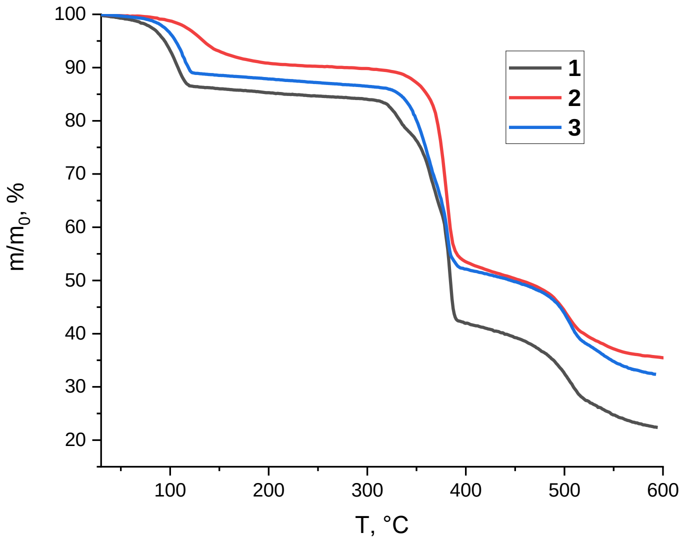

Figure S5. TG plots for **1 – 3**

Table S1. Composition of mixed-metal samples **4 – 12**.

Com-pound	Y : Eu : Tb ratio		Assumed formula
	Starting*	Determined	$[Ln_2(phen)_2(NO_3)_2(chdc)_2]\cdot 2DMF$, Ln_2 =
**4**	99.9 : 0.1 : 0	99.91 : 0.09 : 0	$Y_{1.998}Eu_{0.002}$
**5**	99 : 1 : 0	99.13 : 0.87 : 0	$Y_{1.983}Eu_{0.017}$
**6**	99.9 : 0 : 0.1	99.90 : 0 : 0.10	$Y_{1.998}Tb_{0.002}$
**7**	99 : 0 : 1	98.96 : 0 : 1.04	$Y_{1.979}Tb_{0.021}$
**8**	90 : 0.25 : 9.75	90.65 : 0.32 : 9.03	$Y_{1.813}Eu_{0.006}Tb_{0.181}$
**9**	92 : 0.1 : 7.9	92.44 : 0.07 : 7.49	$Y_{1.849}Eu_{0.0014}Tb_{0.1496}$
**10**	95 : 1 : 4	92.74 : 2.45 : 4.81	$Y_{1.855}Eu_{0.049}Tb_{0.096}$
**11**	98 : 0.05 : 1.95	97.95 : 0.05 : 2.00	$Y_{1.959}Eu_{0.001}Tb_{0.040}$
**12**	98 : 0.0125 : 1.99	97.97 : 0.01 : 2.02	$Y_{1.9594}Eu_{0.0002}Tb_{0.0404}$

*at summary metal salts amount of 0.20 moles, similar to the described one in synthetic methods of **1 – 3**.

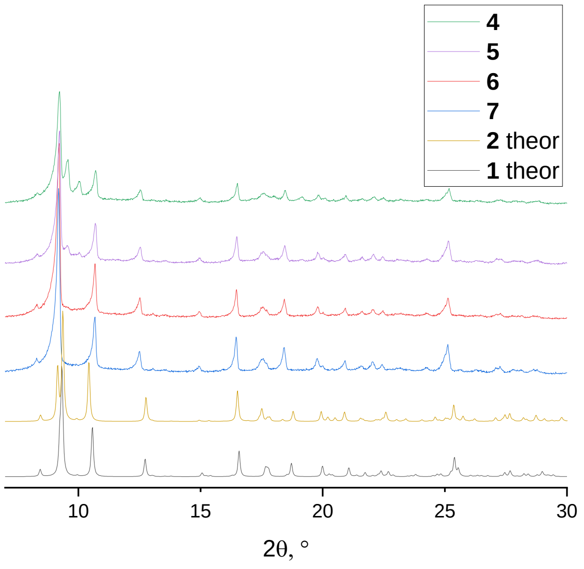

Figure S6. Experimental PXRD patterns for bimetallic samples.

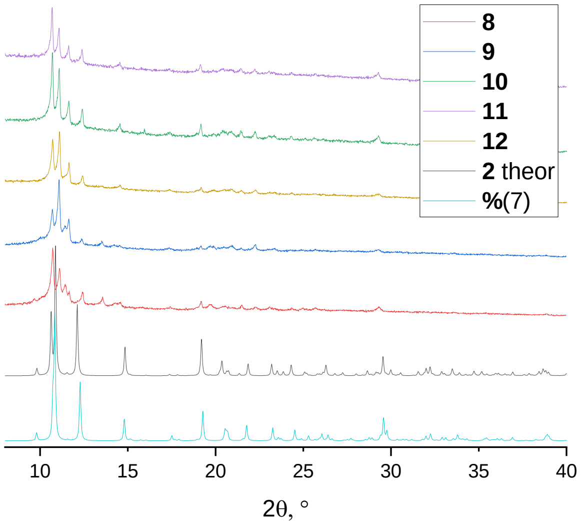

Figure S7. Experimental PXRD patterns for trimetallic samples.

Table S2. Luminescence quantum yields for mixed-metal compositions.

Compound	QY, % at λ_{ex}, nm		
	310	330	350
**4**	6.2	7.3	0.6
**5**	1.7	2.0	5.5
**6**	2.8	3.6	3.2
**7**	3.2	4.0	10
**8**	43	44	71
**9**	39	41	84
**10**	31	30	30
**11**	-	4.5	-
**12**	-	0.1	-

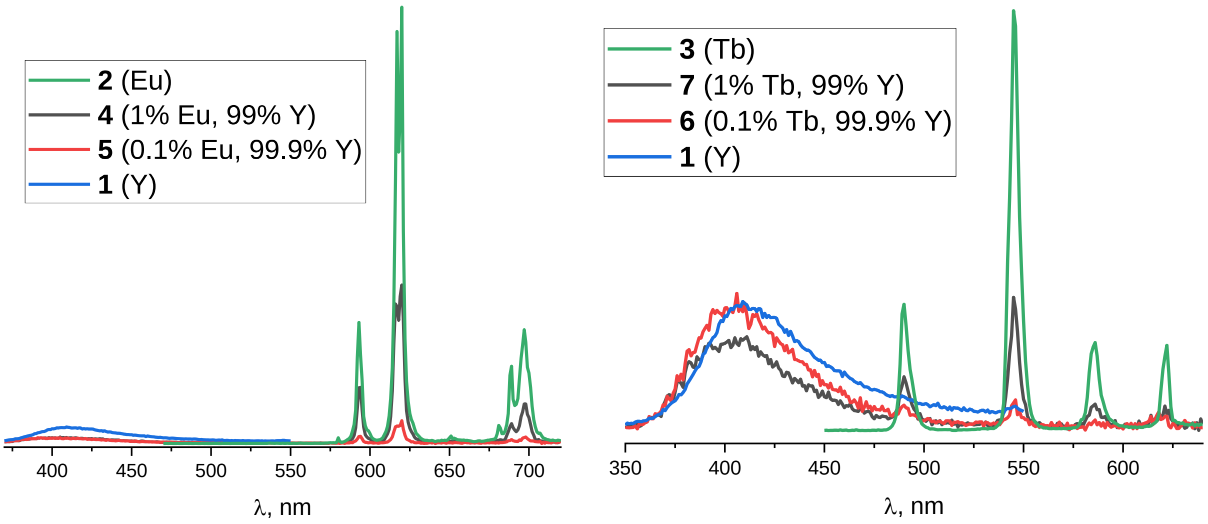

(a) (b)

Figure S8. Luminescence spectra of bimetallic samples **4 – 7** compared to monometallic **1 – 3**. For **2** λ_{ex} = 340 nm. For the rest of the samples λ_{ex} = 330 nm.

Table S3. CIE 1931 coordinates shown on Fig.2b.

Compound	x	y
**1**	0.363	0.581
**2**	0.154	0.039
**3**	0.657	0.339
**4**	0.163	0.082
**5**	0.202	0.196
**6**	0.397	0.2
**7**	0.576	0.302

Table S4. CIE 1931 coordinates shown on Fig.3.

Com-pound	**(x,y) at the matched λ_{ex}, nm**					
	**310**	**330**	**350**	**370**	**380**	**390**
**8**	(0.448,0.450)	(0.493,0.426)	(0.490,0.426)	(0.375,0.340)	**(0.310,0.312)**	(0.283,0.322)
**9**	(0.391,0.462)	(0.428,0.446)	(0.426,0.444)	**(0.335,0.326)**	(0.293,0.295)	(0.281,0.313)
**10**	(0.587,0.351)	(0.615,0.341)	(0.613,0.340)	(0.509,0.322)	(0.403,0.320)	**(0.347,0.335)**
**11**	-	(0.400,0.417)	-	(0.267,0.331)	-	-
**12**	-	(0.384,0.453)	-	(0.289,0.373)	-	-

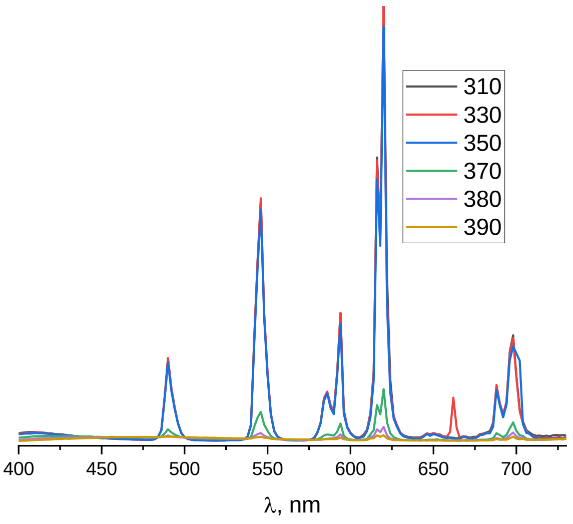

(a)

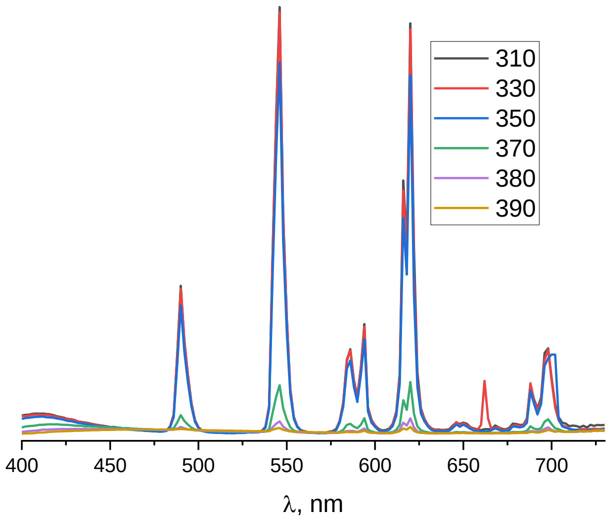

(b)

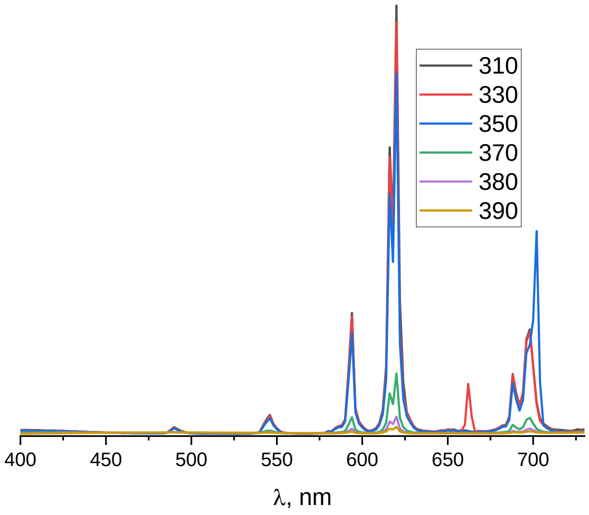

(c)

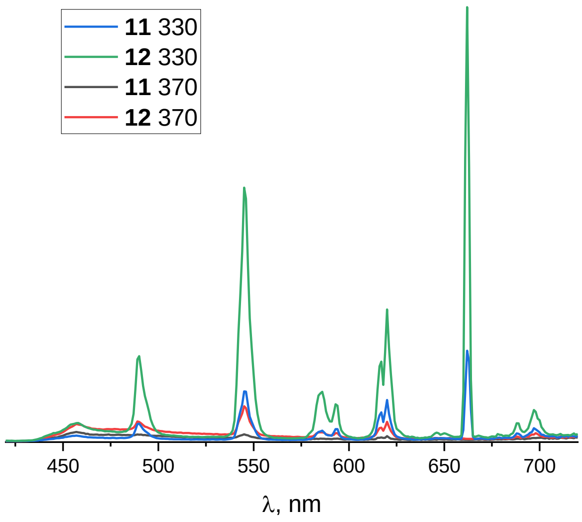

(d)

Figure S9. Luminescence spectra of trimetallic samples **8** (a)**, 9** (b)**, 10** (c) **and 11-12** (d) at different excitation wavelengths.

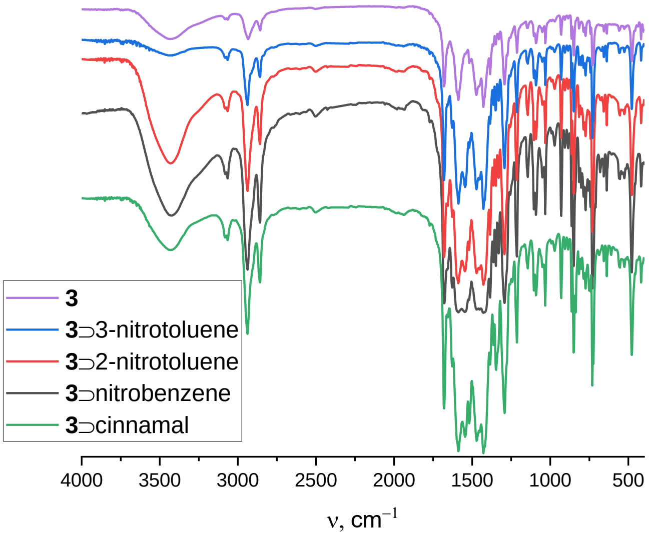

Figure S10. IR spectra for the samples of **3** immersed in liquid nitroaromatics.

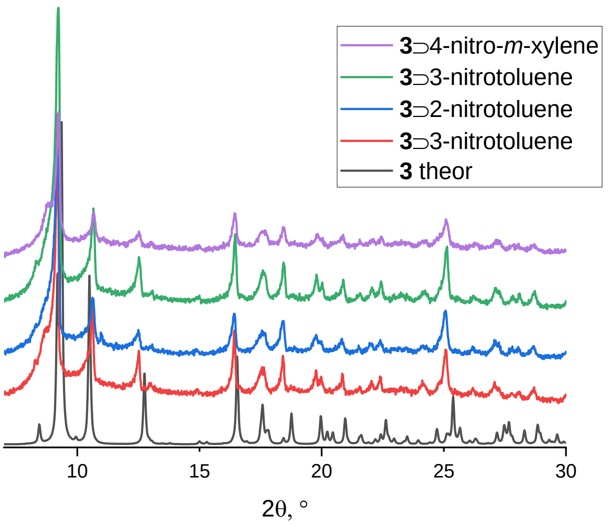

Figure S11. PXRD patterns for the samples of **3** immersed in liquid nitroaromatics compared to the theoretical one for **3**.

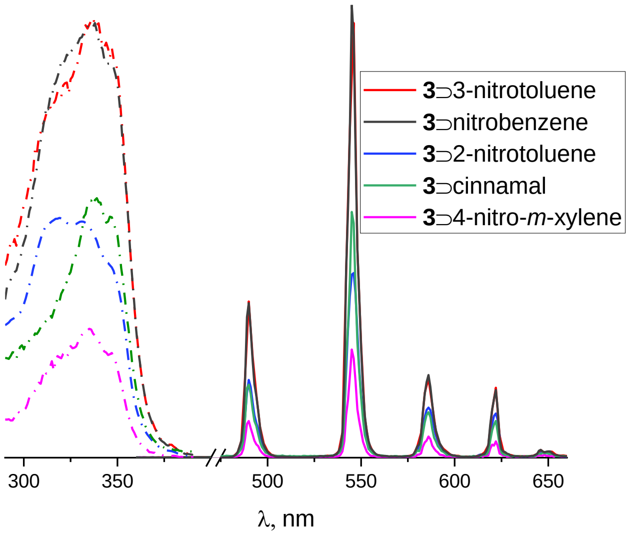

Figure S12. Excitation and emission spectra for the samples of **3** immersed in liquid nitroaromatics and cinnamal.

Table S5. Quantum yields for the samples of **3** immersed in liquid nitroaromatics and cinnamal at λ_{ex} = 330 nm compared to as-synthesized **3**.

Sample	QY, %
**3**	13.5
**3⊃nitrobenzene**	4.3
**3⊃2-nitrotoluene**	2.5
**3⊃3-nitrotoluene**	4.4
**3⊃4-nitro-m-xylene**	1.1
**3⊃cinnamal**	2.5

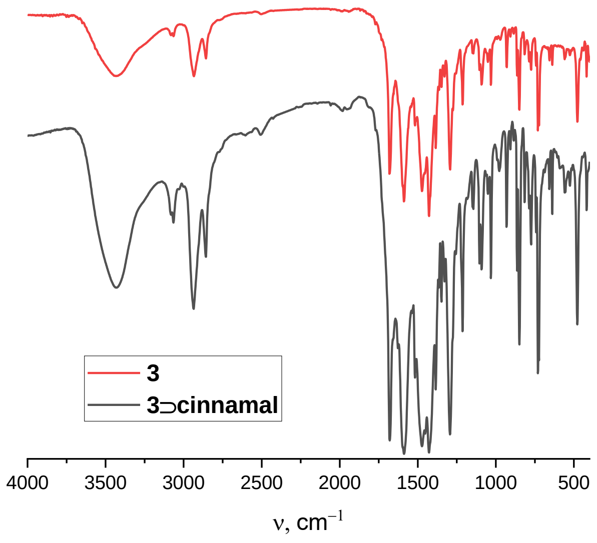

Figure S13. IR spectra for the samples **3** and **3⊃cinnamal**.

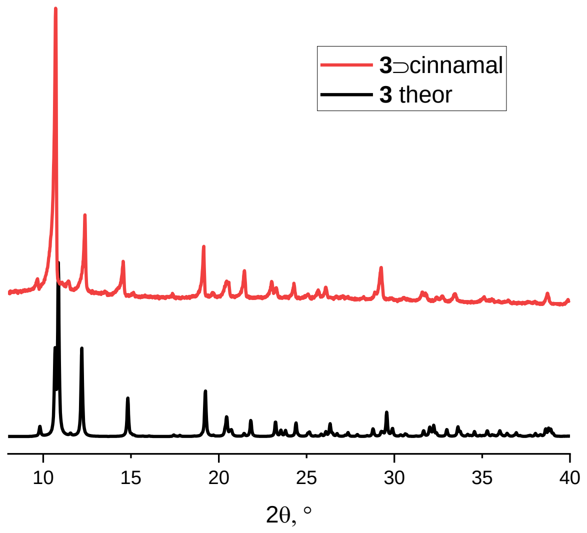

Figure S14. Experimental PXRD pattern for **3⊃cinnamal** compared to the theoretical one for **3**.

Single crystal X-Ray diffraction analysis details. Diffraction data for single crystals of **1** were obtained on an automated Agilent Xcalibur diffractometer equipped with an area AtlasS2 detector (graphite monochromator, λ(MoKα) = 0.71073 Å, ω-scans with a step of 1°). Integration, absorption correction, and determination of unit cell parameters were performed using the CrysAlisPro program package [1]. Diffraction data for single crystals of **2** and **3** were collected on the 'Belok' beamline (λ = 0.7927 Å) of the National Research Center 'Kurchatov Institute' (Moscow, Russian Federation) using a Rayonix SX165 CCD detector. The data were indexed, integrated and scaled, absorption correction was applied using the XDS program package [2]. The structures were solved by dual space algorithm (SHELXT [3]) and refined by the full-matrix least squares technique (SHELXL [4]) in the anisotropic approximation (except hydrogen atoms). Positions of hydrogen atoms of organic ligands were calculated geometrically and refined in the riding model. The crystallographic data and details of the structure refinements are summarized in Table S4. CCDC 2098708–2098710 contain the supplementary crystallographic data for this paper. These data can be obtained free of charge from The Cambridge Crystallographic Data Center at https://www.ccdc.cam.ac.uk/structures/.

Table S6. Crystal data and structure refinement for **1 – 3.**

Compound	**1**	**2**	**3**
Chemical formula	$C_{46}H_{50}N_8O_{16}Y_2$	$C_{46}H_{50}Eu_2N_8O_{16}$	$C_{46}H_{50}N_8O_{16}Tb_2$
M, g/mol	1148.76	1274.86	1288.78
Crystal system	Monoclinic	Monoclinic	Monoclinic
Space group	$P2_1/n$	$P2_1/n$	$P2_1/n$
Temperature, K	130	100	100
a, Å	12.4769(7)	12.337(3)	12.394(3)
b, Å	16.7224(7)	16.950(3)	16.841(4)
c, Å	12.6578(6)	12.760(3)	12.717(3)
β (°)	112.883(6)	112.95(3)	112.90(3)
V (Å^3)	2433.1(2)	2457.1(11)	2445.2(11)
Z	2	2	2
F(000)	1176	1272	1280
D(calc.), g/cm^3	1.568	1.723	1.750
μ, mm^{-1}	2.45	3.46	3.92
Crystal size, mm	0.32 × 0.21 × 0.14	0.15 × 0.03 × 0.03	0.17 × 0.15 × 0.09
θ range for data collection, deg.	$3.47 \leq \theta \leq 25.24$	$2.55 \leq \theta \leq 28.40$	$2.17 \leq \theta \leq 28.40$
Radiation source	fine-focus sealed tube	synchrotron	synchrotron
T_{min}, T_{max}	0.926, 1.000	0.692, 1.000	0.773, 1.000
No. of reflections: measured / independent / observed [$I > 2\sigma(I)$]	11232 / 4438 / 3927	13686 / 4467 / 4175	16000 / 4320 / 4003
R_{int}	0.0237	0.0608	0.1660
Index ranges	$-14 \leq h \leq 15$, $-20 \leq k \leq 17$ $-15 \leq l \leq 14$	$-12 \leq h \leq 14$ $-20 \leq k \leq 20$ $-15 \leq l \leq 15$	$-14 \leq h \leq 14$ $-19 \leq k \leq 19$ $-15 \leq l \leq 15$
Final *R* indices [$I > 2\sigma(I)$]	$R_1 = 0.0276$ $wR_2 = 0.0683$	$R_1 = 0.0342$ $wR_2 = 0.0808$	$R_1 = 0.0547$ $wR_2 = 0.1338$
R indices (all data)	$R_1 = 0.0342$ $wR_2 = 0.0707$	$R_1 = 0.0363$ $wR_2 = 0.0823$	$R_1 = 0.0597$ $wR_2 = 0.1371$
Goodness-of-fit on F^2	1.055	1.049	1.047
Largest diff. peak / hole, *e*/Å^3	0.73, −0.51	0.96, −1.49	1.21, −1.37

References

1. CrysAlisPro 1.171.38.46. Rigaku Oxford Diffraction: The Woodlands, TX, USA, 2015

2. Kabsch, W. XDS. *Acta Crystallogr.* **2010**, *D66*, 125.

3. Sheldrick, G.M. SHELXT - Integrated space-group and crystal-structure determination. *Acta Crystallogr*. **2015**, *A71*, 3.

4. Sheldrick, G.M. Crystal structure refinement with SHELXL. *Acta Crystallogr*. **2015**, *C71*, 3.
